# Removal of 3-methylindole by lactic acid bacteria *in vitro*

**DOI:** 10.3892/etm.2013.1251

**Published:** 2013-08-06

**Authors:** XIAO MENG, ZHI-FEI HE, HONG-JUN LI, XIN ZHAO

**Affiliations:** 1 College of Food Science, Southwest University, Chongqing 400715;; 2 Department of Biological and Chemical Engineering, Chongqing University of Education, Chongqing 400067, P.R. China;; 3 Department of Food Science and Nutrition, Pusan National University, Busan 609-735, Republic of Korea

**Keywords:** removal, 3-methylindole, lactic acid bacteria

## Abstract

3-Methylindole (3MI) is a substance with an unpleasant odor that is found in intact male pigs and is known to negatively affect consumers of pork. The growth of four strains of lactic acid bacteria [*Lactobacillus brevis* 1.12 (*L. brevis* 1.12), *L. plantarum* 102, *L. casei* 6103 and *L. plantarum* ATCC8014] in incubation medium with 3MI was studied. The four strains were tested for their ability to remove 3MI from the medium. The growth of *L. brevis* 1.12 remained steady as the levels of 3MI increased 3MI from 0.2 to 1.0 *μ*g/ml. The 3MI removal ability of *L. brevis* 1.12 was the strongest among the four strains, and the highest removal rate was 65.35±0.3% in 1 ml incubation medium containing 1.0 *μ*g/ml 3MI for 120 h. Furthermore, the supernatant fluid of the fermentation broth of *L. brevis* 1.12 had a stronger ability to remove 3MI than cell pellets and cell extracts and the removal rate was 14.4±0.3% in 24 h. Further results indicate that the mode of removal of 3MI was not through the physical binding of cells by *L. brevis* 1.12.

## Introduction

3-Methylindole (3MI) is a substance with an unpleasant odor (1–4) that is produced by the tryptophan removal process in the caecum and colon of intact male pigs (5,6). It has been reported that consumers are able to detect the negative odor when the level of 3MI is >0.21 mg/kg (7).

In numerous countries, the castration of male piglets is common practice to remove the odor. However, intact male pigs have demonstrated a superior performance compared with barrows, due to better carcass traits, lean meat percentage and feed efficiency. Furthermore, the surgical castration of male piglets not only diminishes the benefits of intact male pigs, but also increases concerns about animal welfare (1). Surgical castration is not performed in Australia, the United Kingdom and Ireland. In Norway, a total ban on castration was initiated in January 2009 (8). However, if intact male pigs are to be used for pork production, the 3MI level must be substantially reduced. At present, a number of microbial methods have been used to investigate the problem *in vitro*. Compared with physical and chemical methods, biological techniques are preferable due to the economical advantages and low possibility of byproduct generation. Various bacterial strains have been applied to reduce the levels of harmful substances for a number of years. *Ochrobactrum intermedium* DN2 has been used to degrade nicotine in tobacco waste extracts and the average degradation rate of nicotine in a 30 l fed-batch culture was 140.5 mg/l/h (9). In addition, *Shinella zoogloeoides* BC026 has been identified to reduce pyridine levels, resulting in a degradation rate of 1,806 mg/l pyridine in 45.5 h (10). Moreover, *Bacillus odysseyi* SUK3, *Morganella morganii* SUK5 and *Proteus species* SUK7 have been shown to decolorize Reactive Blue 59 (50 mg/l) completely within 60, 30 and 24 h, respectively (11). With regard to 3MI degradation, Kohda *et al* (12) identified that 0.05% (w/v) 3MI may be degraded by a type of *Clostridium* from the feces of pigs in under 4 weeks with a removal rate of up to 32.18%. Yin *et al* (13) demonstrated that 2.5 mmol/l 3MI may be reduced by *Pseudomonas aeruginosa* (extracted from the sediment of lapacho wood) in 3 days and the time was extended as the 3MI concentration increased from 2.5 to 3.5 mmol/l. Additionally, Gu and Berry (14) indicated that 1–1.5 *μ*mol/l 3MI was reduced in 36 days by a bacterial colony that produced methane. Furthermore, Gu *et al* (15) reported that 3MI may be completely degraded by sulfate-reducing bacteria.

The objectives of the present study were to investigate the growth of lactic acid bacteria [*Lactobacillus brevis* 1.12 (*L. brevis* 1.12), *L. plantarum* 102, *L. casei* 6103 and *L. plantarum* ATCC8014] in culture medium with varied concentrations of 3MI, and to explore the correlation between the levels of 3MI and the 3MI removal ability of the lactic acid bacteria during the fermentation process *in vitro*.

## Materials and methods

### Strains and medium

*L. brevis* 1.12, *L. casei* 6103 and *L. plantarum* ATCC8014 were purchased from China Center of Industrial Culture Collection (Beijing, China). *L. plantarum* 102 was obtained from American Type Culture Collection (Manassas, VA, USA). MRS broth (Oxoid Ltd., Basingstoke, UK) was used as the culture medium for the lactic acid bacteria.

### Chemicals and reagents

3MI, acetonitrile and methanol of high-performance liquid chromatography (HPLC) grade were purchased from Sigma-Aldrich (St. Louis, MO, USA). Phosphate-buffered saline (PBS; Sigma-Aldrich) was of analytical grade. All remaining chemicals were of biological and analytical reagent grades, and were obtained from Kelong Chemical Reagent Factory (Chengdu, China).

### Effects of 3MI on the growth of lactic acid bacteria

The 3MI standard solutions contained 0.0, 0.2, 0.4, 0.6, 0.8 and 1.0 g 3MI standard substance dissolved in 10 ml absolute ethyl alcohol (w/v; 0.00, 0.02, 0.04, 0.06, 0.08 and 0.10 g/ml, respectively). The bacterial colonies of *L. brevis* 1.12, *L. plantarum* 102, *L.* casei 6103 and *L. plantarum* ATCC8014 were suspended in 10 ml 0.75% (w/v) physiological saline. Subsequently, 2.5% (v/v) suspension liquid and 1 ml 3MI solution were mixed with 100 ml MRS medium for 72 h at 37°C. The optical density (OD) value at 600 nm was detected every 2 h by a spectrophotometer (722-spectrophotometer; Tairui Instrument Co., Ltd., Chongqing, China) (16,17).

### Removal of 3MI from the MRS broth by fermentation of lactic acid bacteria strains

3MI standard substance (0.0, 0.2, 0.4, 0.6, 0.8 and 1.0 g) was dissolved in 10 ml absolute ethyl alcohol to provide 3MI standard solutions (0.00, 0.02, 0.04, 0.06, 0.08 and 0.10 g/ml, respectively). Bacterial colonies of *L. brevis* 1.12, *L. plantarum* 102, *L. casei* 6103 and *L. plantarum* ATCC8014 were suspended in 10 ml 0.75% (w/v) physiological saline. 3MI solution (1 ml) and 2.5% (v/v) suspension liquid were mixed in 100 ml MRS medium for 120 h at 37°C. The sample treatment method for HPLC was as follows: The fermentation broth was centrifuged at 9000 × g for 10 min; 1 ml of supernatant was mixed with 9 ml acetonitrile:ultrapure water (75:25, v/v); and the intermixture was filtered through an organic phase filter of 0.45 *μ*m (Frisenette ApS Co., Ebeltoft, Denmark) and loaded into a 1.5 ml screw-thread bottle (16,17). In the present study, the removal rate of 3MI (%) was the response value, which was calculated using the following equation: Removal rate (%) = (A-B)/Ax100; where A is the initial level of 3MI (ng/ml) and B represents the residual level of 3MI (ng/ml).

### Removal of 3MI by the supernatant fluid of fermentation, cell pellets and extracts of lactic acid bacteria

An activated culture of lactic acid bacteria (10 ml) was centrifuged (9,000 × g, 5 min, 5°C). The supernatant fluid of fermentation was collected, and the resultant cell pellets were washed twice with 10 ml sterile PBS (0.01 M, pH 7.4) and suspended in 10 ml sterile PBS. The cell pellet suspension (10 ml) was disintegrated (400 W every 5 sec for 30 min) in an ice-water bath by an ultrasonic cell disintegrator (Branson Sonifier 450; Branson Ultrasonics Corp., Danbury, CT, USA). The disintegrated cell suspension was centrifuged (9,000 × g, 5 min, 5°C) and the supernatant (cell extract) was collected. 3MI was added to the solutions of supernatant fluid of fermentation, suspensions of cell pellets and cell extracts of lactic acid bacteria, to an initial 3MI concentration of 1.0 *μ*g/ml. Sterile PBS containing 1.0 *μ*g/ml 3MI was used as the control. The suspensions were incubated at 37°C for 24 h, centrifuged (9,000 × g, 5 min, 5°C) and the supernatant fluids were filtered through a 0.45-*μ*m filter and stored at 4°C prior to analysis (18,19).

### Removal of 3MI from PBS by viable heat-, acid- and alkali-treated cells and the supernatant fluid of lactic acid bacteria

Activated culture of *L. brevis* 1.12 (10 ml) was centrifuged (9,000 × g, 5 min, 5°C) and the cell pellets were washed twice with 10 ml sterile PBS (0.01 M, pH 7.4). Cells of *L. brevis* 1.12 were treated by the following methods: heating (100°C for 30 min, incubated at 37°C for 24 h), acid treatment (1 M HCl, incubated at 37°C for 24 h) and alkali treatment (1 M NaOH, incubated at 37°C for 24 h). Following these treatments, the suspensions were centrifuged (9,000 × g, 5 min, 5°C) and the supernatants were removed. The resultant cell pellets were washed twice with 10 ml sterile PBS and then suspended in 10 ml sterile PBS. Sterile PBS containing 1.0 *μ*g/ml 3MI was used as the control. The removal of 3MI was tested as previously described. The supernatant fluid of fermentation was cryodesiccated and treated by the same method used for cells of *L. brevis* 1.12 (20,21).

### Analysis of 3MI by HPLC

For HPLC, an LC-20A system (Shimadzu Co., Kyoto, Japan) was used consisting of a SIL-10ADvp injector with a 100 *μ*l loop and two LC-10 ADvp HPLC pumps. The detector used was a RF-20A fluorometer and data were collected with an LC solution integrator. The column was Hypersil (ODS-2, 5-*μ*m particle size; length, 200 mm; tubing I.D, 4.6 mm; Elite Analytical Instrument Co., Ltd., Dalian, China) and operated at ambient temperature. The mobile phase consisted of acetonitrile:ultrapure water (60:40, v/v) and the flow rate was 1.0 ml/min. The detection was carried out by measuring the fluorescence with the following wavelengths: excitation at 281 nm and emission at 353 nm. The volume of the injected sample was 10 *μ*l (22).

### Statistical analysis

All data are expressed as the mean ± standard deviation of triplicate assays. Simple linear regressions for 3MI standard solutions with different gradient concentrations and the growth of lactic acid bacteria in the MRS medium with 3MI were calculated using Microsoft Excel 2007. Statistical analyses for the removal rate of 3MI (%) were carried out using PASW statistics (formerly SPSS), version 18.0 (IBM SPSS, Inc., Chicago, IL, USA).

## Results

### Effects of 3MI on the growth of lactic acid bacteria

**Figure 1.**
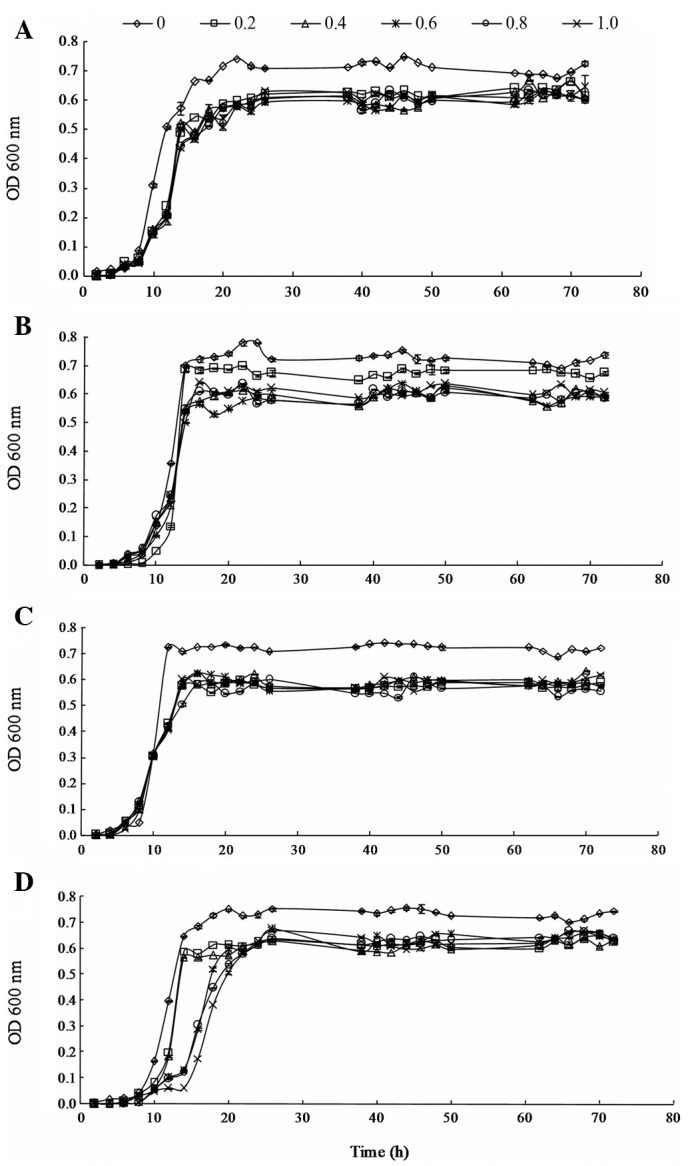
Growth of lactic acid bacteria in MRS medium with 0.2-1.0 *μ*g/ml 3MI during 72 h of incubation at 37°C. Lactic acid bacteria in the MRS broth without 3MI was used as the control. Different symbols represent the various 3MI levels. The growth of (A) *Lactobacillus brevis* 1.12, (B) *L. plantarum* 102, (C) *L. casei* 6103 and (D) *L. plantarum* ATCC8014. 3MI, 3-methylindole; OD, optical density.

The growth responses of lactic acid bacteria (*L. brevis* 1.12, *L. plantarum* 102, *L. casei* 6103 and *L. plantarum* ATCC8014) to different concentrations of 3MI (from 0.2 to 1.0 *μ*g/ml) are shown in Fig. 1.

The effects of 3MI on the growth of L. *brevis* 1.12 were not significant during the lag phase. However, the growth of *L. brevis* 1.12 was inhibited by 3MI during the logarithmic phase. During the stationary phase, the OD values of *L. brevis* 1.12 in the MRS medium containing 3MI were lower than such values of *L. brevis* 1.12 in MRS medium without 3MI. However, the growth of *L. brevis* 1.12 was steady as the level of 3MI increased from 0.2 to 1.0 *μ*g/ml in the stationary phase.

The effects of 3MI on the growth of *L. plantarum* 102 were not significant at the lag and logarithmic phases. In addition, the results showed that the growth of *L. plantarum* 102 was not inhibited by 3MI at the two phases. During the stationary phase, the growth of *L. plantarum* 102 was restrained in the presence of increasing levels of 3MI (from 0.2 to 1.0 *μ*g/ml) and significant differences between the effects of the various levels of 3MI on the growth of *L. plantarum* 102 were observed.

The effects of 3MI on the growth of *L. casei* 6103 were not significant at the lag and logarithmic phases; however, growth was slower than that of the control group at 10–12 h in the logarithmic phase. At the stationary phase, the growth of *L. casei* 6103 was restrained in the MRS medium with 0.2 *μ*g/ml 3MI, but the OD value was steady as the level of 3MI increased from 0.2 to 1.0 *μ*g/ml. The results suggest that the growth of *L. casei* 6103 was inhibited by 3MI at the stationary phase; however, the difference in growth among the varied levels of 3MI was not marked.

The effects of 3MI on the growth of *L. plantarum* ATCC8014 were significant at the logarithmic phase, with slower growth compared with that of the control. However, during the stationary phase, the growth was steady with increasing concentrations of 3MI from 0.2 to 1.0 *μ*g/ml.

### 3MI removal during the fermentation of lactic acid bacteria

The relationship between the concentration of 3MI and the removal ability of the four strains during the fermentation process was also studied. The results indicate that the levels of 3MI decreased during the fermentation process in all four strains and the removal rate increased as the incubation time increased from 24 to 120 h. Differences among the four strains were significant, as shown in Table I. *L. brevis* 1.12 indicated the strongest ability to remove 3MI compared with the other strains. The removal rate increased as the incubation time was extended from 24 to 120 h; however, the effects of different 3MI levels on the ability of *L. brevis* 1.12 to remove MI were not significant, as the removal rate was steady with increasing levels of 3MI from 0.2 to 1.0 *μ*g/ml; the highest removal rate was 65.35±0.3% in the fermentation fluid of *L. brevis* 1.12 with 1.0 *μ*g/ml 3MI in 120 h. For *L. plantarum* 102, *L. casei* 6103 and *L. plantarum* ATCC8014, the effects of the different levels of 3MI on the removal ability of the three strains were significant. The ability of the three strains to remove 3MI decreased as the 3MI levels increased from 0.2 to 1.0 *μ*g/ml, and *L. plantarum* 102 and *L. plantarum* ATCC8014 were more sensitive to 3MI when compared with *L. casei* 6103. The removal ability of *L. plantarum* 102 and *L. plantarum* ATCC8014 was sensitive to 0.4 and 0.8 *μ*g/ml 3MI, respectively. The removal rates were 28.54±0.2 and 33.23±0.9% in the fermentation fluid of *L. plantarum* 102 and *L. plantarum* ATCC8014 with 1.0 *μ*g/ml 3MI, respectively, in 120 h.

### Mode of removal

**Figure 2.**
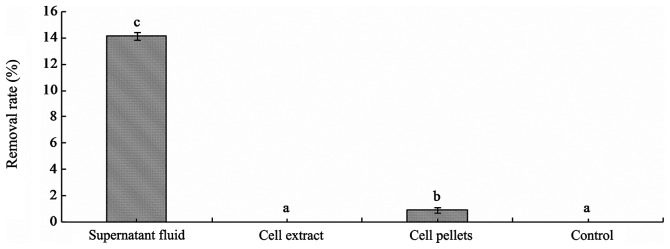
Removal rate of 3MI following incubation with the supernatant fluid of fermentation, suspension of cell pellets and cell extracts of *L. brevis* 1.12 at 37°C for 24 h. Sterile PBS containing 1.0 *μ*g/ml 3MI was used as the control. Values of columns labeled with different letters were significantly different (P<0.05). 3MI, 3-methylindole; PBS, phosphate-buffered saline.

The concentrations of 3MI following incubation with the supernatant fluid of fermentation, the suspension of cell pellets and cell extracts of *L. brevis* 1.12 at 37°C for 24 h were detected by HPLC. The results showed that the 3MI removal ability of the supernatant fluid of fermentation broth was the strongest. 3MI was removed by the supernatant fluid of fermentation with a removal rate of 14.4±0.3% at 37°C for 24 h. 3MI was removed by cell pellets of *L. brevis* 1.12 (0.88±0.2%) (Fig. 2), but 3MI was not detected in the PBS eluent. The results suggest that the removal mode of 3MI was not through the physical binding of cells by *L. brevis* 1.12. Furthermore, the results showed that the removal rates of 3MI in the suspensions following incubation with heat-, acid- and alkali-treated cells decreased significantly, and the removal ability of *L. brevis* 1.12 was inhibited under these methods (Table II). The results also confirmed that the removal mode for 3MI was not via physical binding.

## Discussion

In the present study, the results suggest that the four strains of lactic acid bacteria are more sensitive to 3MI than previously investigated microorganisms from other studies regarding the effects of 3MI on microorganisms. The growth of all four strains was inhibited by low levels of 3MI (0.2 *μ*g/ml). However, Dreizen and Spies (23) identified that the growth of *L. acidophilus* was completely restricted by 400 *μ*g/ml 3MI, but growth occurred when the 3MI concentration was decreased from 400 to 100 *μ*g/ml. In addition, Tittlser *et al* (24) demonstrated that the growth of 25 species of Gram-negative bacteria extracted from the intestinal tract was inhibited when the 3MI concentration was 330 *μ*g/ml. Furthermore, Kohda *et al* (12) identified that the growth of *Clostridium* was steady in 100–300 *μ*g/ml 3MI solution; however, the growth of certain clostridia was prevented in 50 *μ*g/ml 3MI solution. In the present study, the growth of the four strains was prevented in the incubation environment with 0.2 *μ*g/ml 3MI and the tolerance levels for 3MI concentration were lower than those identified in the previously mentioned studies.

Furthermore, the ability of the four strains to degrade 3MI was compared with results from previous studies. The results showed that the 3MI removal ability of the four strains was stronger than that of the microorganisms investigated in the studies by Gu *et al* (15) and Li *et al* (25). Gu *et al* (15) identified that 3MI may be degraded by marine anaerobic microorganisms for 30 days. Additionally, Li *et al* (22) indicated that 2.0 mmol/l 3MI may be degraded by *Pseudomonas putida* LPC24 in <30 days. In the present study, the degradation ability of *L. brevis* 1.12 was the strongest among the bacteria tested, with a degradation rate for 1.0 *μ*g/ml 3MI of 65.35±0.3% in 5 days. However, the degradation time was longer than that in the study by Yin *et al* (13). The study suggested that 2.5 mmol/l 3MI may be reduced by *Pseudomonas aeruginosa* (extracted from the sediment of lapacho wood) in 3 days and the time extended with increased 3MI concentration from 2.5 to 3.5 mmol/l (13). The different results may be due to significant differences among the microorganisms tested.

The results of the present study demonstrated that 3MI may be degraded by the supernatant fluid of fermentation and suspension of cell pellets; however, 3MI was not detected in the eluent of cell pellets. This suggests the key substance responsible for the degradation of 3MI exists in the supernatant fluid of the fermentation broth and that the mode of 3MI removal was not through the physical binding of cells by *L. brevis* 1.12. However, in the present study, the removal mechanism of 3MI during the fermentation process of *L. brevis* 1.12 was not studied. The removal mechanism of 3MI in certain microorganisms and the liver of entire pigs have been demonstrated in previous studies. Gu *et al* (15) identified that 3MI may be degraded by marine anaerobic microorganisms, and the mechanism included two steps of oxidation accomplished by hydroxylation and then dehydrogenation at the 2- and 3-positions sequentially prior to the cleavage of the pyrrole ring between the 2- and 3-positions. The 3MI degradation mechanism in pig liver is usually conducted in two steps, phase I and II (26). Phase I consists of an oxidation of the compound, usually catalyzed by cytochrome P450 (CYP450) enzymes, while phase II is conducted by a more diverse group of enzymes and consists of conjugation with a hydrophilic group, such as by glucuronidation, sulfoconjugation or glucosidation. The outcome of phase I and/or II metabolism is often the elimination of the compound by excretion (26). In pigs, the phase I metabolism of 3MI is mainly mediated by hepatic CYP1A2, CYP2A and CYP2E1 (27). Diaz and Squires (28) indicated that 3MI is metabolized by the CYP450 system in the lungs and liver of ruminants, rodents and humans. Squires and Lundström (26) demonstrated that a similar system may be involved in 3MI metabolism in pigs and a particular CYP2E1 was indicated as the major enzyme responsible for metabolic breakdown of 3MI in the liver. Moreover, Chen *et al* (8) identified that a dietary supplement of raw potato starch reduced the levels of 3MI. Furthermore, the addition of fructooligosaccharide to pig fecal slurries significantly reduced 3MI levels but not indole synthesis from tryptophan. In future studies, the supernatant fluid of the fermentation broth with 3MI will be detected using HPLC to analyze whether new metabolites are produced and to study the mechanism of 3MI degradation by *L. brevis* 1.12.

In conclusion, the present study used various *in vitro* experimental methods to investigate the growth of lactic acid bacteria in the presence of 3MI, the 3MI removal ability of four strains of bacteria during the fermentation process and the removal mode of 3MI by *L. brevis* 1.12. The results demonstrated that the growth of all four strains was inhibited by 3MI and the 3MI removal ability of *L. brevis* 1.12 was the strongest. The 3MI removal rate of *L. brevis* 1.12 was 65.35±0.3% from the incubation medium of 1 ml 1.0 *μ*g/ml 3MI in 120 h. Furthermore, this study demonstrated that the mode of 3MI removal was not through the physical binding of cells by *L. brevis* 1.12.

## Figures and Tables

**Table I.** Effects of the level of 3MI on the 3MI removal ability of lactic acid bacteria.

Concentration of 3MI (*μ*g/ml)	Incubation time (h)	Removal rate (%)			
		*L. brevis* 1.12	*L. plantarum* 102	*L. casei* 6103	*L. plantarum* ATCC8014
0.2	24	15.71±0.20^a^	11.47±0.40^b^	9.20±0.10^c^	11.55±0.20^b^
	48	30.62±0.10^b^	39.77±0.10^a^	21.39±0.40^c^	30.50±0.10^b^
	72	57.36±0.60^a^	53.33±0.30^c^	54.23±1.50^b^	50.27±0.30^d^
	96	62.64±0.10^a^	57.28±0.10^c^	60.30±0.20^b^	52.09±0.10^d^
	120	67.63±0.20^a^	60.79±0.10^c^	61.53±1.20^b^	56.75±0.10^d^
0.4	24	15.36±0.10^a^	9.46±0.30^b^	6.28±0.60^c^	9.50±0.20^b^
	48	29.81±0.10^a^	25.38±0.10^b^	20.72±0.50^c^	17.90±0.20^d^
	72	57.20±0.10^a^	46.21±0.50^c^	53.55±0.40^b^	26.64±0.90^d^
	96	62.12±0.10^a^	53.51±0.50^c^	57.14±0.20^b^	27.32±0.20^d^
	120	67.87±0.20^a^	55.02±0.70^c^	59.16±0.10^b^	35.41±0.30^d^
0.6	24	15.13±0.60^a^	2.65±0.40^d^	6.21±0.50^c^	7.70±0.80^b^
	48	30.93±0.20^a^	19.38±0.30^c^	20.16±0.50^b^	17.93±0.10^d^
	72	57.52±0.20^a^	40.23±0.60^c^	46.89±0.60^b^	23.96±0.10^d^
	96	61.76±0.40^a^	46.72±0.10^c^	50.32±0.60^b^	26.73±0.70^d^
	120	67.61±0.30^a^	47.51±0.20^c^	59.14±0.20^b^	35.20±0.20^d^
0.8	24	16.09±0.10^a^	1.05±0.10^d^	4.21±0.50^c^	8.12±0.30^b^
	48	31.23±0.20^a^	12.44±0.50^d^	17.21±0.80^b^	15.53±0.40^c^
	72	57.66±0.20^a^	33.35±0.30^c^	46.40±0.40^b^	22.35±0.10^d^
	96	61.63±0.10^a^	35.64±0.20^c^	52.18±0.60^b^	23.27±0.60^d^
	120	67.12±0.10^a^	35.68±0.30^c^	52.76±0.10^b^	35.71±0.10^c^
1.0	24	17.43±0.20^a^	0.88±0.10^d^	3.48±0.26^c^	5.50±0.40^b^
	48	31.68±0.20^a^	10.62±0.20^d^	16.43±0.20^b^	19.05±0.90^c^
	72	56.72±0.60^a^	29.60±0.20^c^	39.32±0.30^b^	25.42±0.50^d^
	96	61.55±0.60^a^	29.63±0.10^c^	51.78±0.20^b^	26.16±0.50^d^
	120	65.35±0.30^a^	28.54±0.20^d^	52.15±0.30^b^	33.23±0.90^c^

Values are the mean ± standard deviation of triplicate assays. Values in a row with the same letter are not significantly different (P>0.05) and values in a row with different letters are significantly different (P<0.05). 3MI, 3-methylindole; *L., Lactobacillus.*

**Table II.** Effects of supernatant and cell pellets of *L. brevis* 1.12 on 3MI removal using different treatment methods.

Substance	Removal rate (%)					
	Heat-treated	Acid-treated	Alkali-treated	Non-treated	HCl control	NaOH control
Supernatant	3.98±1.3^a^	6.70±0.7^c^	4.92±1.2^b^	15.27±2.3^d^	−	−
Cell pellets	−	−	−	0.95±1.5	−	−

Incubation time was 24h at 37°C. Values are the mean ± standard deviation of triplicate assays. Values in a row with different letters are significantly different. *L., Lactobacillus.*
